# Express saccades in distinct populations: east, west, and in-between

**DOI:** 10.1007/s00221-017-5094-1

**Published:** 2017-09-27

**Authors:** Paul C. Knox, Felicity D. A. Wolohan, Mai S. Helmy

**Affiliations:** 1 Eye and Vision Science, Institute of Ageing and Chronic Disease, University of Liverpool, William Henry Duncan Building, 6 West Derby St, Liverpool, L7 8TX UK; 2 Department of Psychology, Edge Hill University, Ormskirk, UK; 3 Department of Psychology, University of Menoufia, Shibin El Kom, Egypt

**Keywords:** Saccades, Express saccades, Latency, Gap, Culture

## Abstract

Express saccades are low latency (80–130 ms), visually guided saccades. While their occurrence is encouraged by the use of gap tasks (the fixation target is extinguished 200 ms prior to the saccade target appearing) and suppressed by the use of overlap tasks (the fixation target remains present when the saccade target appears), there are some healthy, adult participants, “express saccade makers” (ESMs), who persist in generating high proportions (> 30%) of express saccades in overlap conditions. These participants are encountered much more frequently in Chinese participant groups than amongst the Caucasian participants tested to date. What is not known is whether this high number of ESMs is only a feature of Chinese participant groups. More broadly, there are few comparative studies of saccade behaviour across large participant groups drawn from different populations. We, therefore, tested an independent group of 70 healthy adult Egyptian participants, using the same equipment and procedures as employed in the previous studies. Each participant was exposed to two blocks of 200 gap, and two blocks of 200 overlap trials, with block order counterbalanced. Results from the Schwartz Value Survey were used to confirm that this group of participants was culturally distinct from the Chinese and Caucasian (white British) groups tested previously. Fourteen percent (10/70) of this new group were ESMs, and the pattern of latency distribution in these ESMs was identical to that identified in the other participant groups, with a prominent peak in the express latency range in overlap conditions. Overall, we identified three modes in the distribution of saccade latency in overlap conditions, the timing of which (express peak at 110 ms, subsequent peaks at 160 and 210 ms) were strikingly consistent with our previous observations. That these behavioural patterns of saccade latency are observed consistently in large participant groups, drawn from geographically, ethnically, and culturally distinct populations, suggests that they relate to the underlying architecture of the saccade system.

**Electronic supplementary material:**

The online version of this article (doi:10.1007/s00221-017-5094-1) contains supplementary material, which is available to authorized users.

## Introduction

“Express” saccades (ES), are reflexive, visually guided saccades that have a latency which is consistent with the minimum afferent and efferent conduction times between the retina and the external oculomotor plant (Fischer et al. 1993; Dorris et al. 1997). Single unit recording in non-human primates has shown that ES production is critically dependant on the superior colliculus where visual responses are transformed into saccade motor commands (Schiller et al. 1987). ES occur when the visual (target onset) response, combined with increased pre-target activity, is sufficient to trigger the brainstem gaze generating circuitry (Dorris et al. 1997; Edelman and Keller 1996; Sparks et al. 2000). When saccade latency distributions are plotted, ES may form a distinct, early mode in the distribution. This pattern, while observed in non-human primates (Fischer and Boch 1983; Schiller et al. 1987), was not always observed in human studies, leading to considerable controversy (Fischer et al. 1993; Kalesnykas and Hallett 1987; Kingstone and Klein 1993; Reuter-Lorenz et al. 1991; Wenban-Smith and Findlay 1991). However, stimulus features and paradigm design influence saccade latency range, the shape of its distribution, and the proportion of ES (Carpenter 2001; Jüttner and Wolf 1992; Marino et al. 2012, 2015).

The proportion of ES is increased by the use of a stimulus in which the fixation point is extinguished prior to the target appearing (the “gap” paradigm) and decreased if the fixation target remains visible when the saccade target appears (the “overlap” paradigm; Mayfrank et al. 1986; Saslow 1967a). It is also increased by “training” (in which participants are exposed to blocks of gap trials over an extended period of time; Bibi and Edelman 2009; Fischer and Ramsperger 1986; Knox and Wolohan 2015), and in certain patient groups (e.g., schizophrenics; Clementz 1996). However, there are some healthy participants, “express saccade makers” (ESMs), who in overlap conditions exhibit a high proportion of express saccades (> 30%). While ESMs had been suggested to be rare (1–5% of the healthy population; Biscaldi et al. 1996), across a number of studies, we found that they occurred much more frequently among Chinese participant groups, making up 20–30% of participants (Amatya et al. 2011; Knox et al. 2012; Knox and Wolohan 2014). In ESMs, saccade latency distributions for overlap tasks were marked by a prominent peak at around 100 ms. While the proportion of ESMs differed markedly between Chinese and Caucasian (white British; WB) participant groups, the characteristics of the ESMs themselves were identical. Training slightly increased the proportion of ES observed in overlap conditions, but it was not possible to train non-ESM participants to produce either the same high proportions of ES observed in ESMs, or the distinct latency distributions observed in ESMs (Knox and Wolohan 2015). This, along with the finding that the proportion of ES is stable over time in the absence of training, and that the high proportion of ESMs in Chinese participant groups is independent of culture and language (Knox and Wolohan 2014), suggests that the pattern of latency observed in ESMs in overlap conditions constitutes an oculomotor behavioural phenotype.

We have only investigated the phenomenon of ES production and patterns of saccade performance in Chinese and WB participants. In the absence of further data, we do not know whether it is the smaller proportion of ESMs in WB participants, or the large proportion among Chinese participants that is unusual. We also do not know whether the distinct multimodal distributions which we have observed in large participant groups is a common feature, or peculiar to the groups which we have studied so far. Therefore, we recruited and tested a third, large, geographically independent group of participants from Shibin El Kom, a city with a population of approximately 300,000 people, located in the Nile delta north of Cairo. We examined the pattern of distribution of saccade latency in these participants and established the proportion of ESMs. Rather than assume that geographic separation necessarily ensures cultural independence, we used the Schwartz Value Survey (SVS) to compare the value systems adopted by different groups as a proxy for culture (Datler et al. 2013; Knox and Wolohan 2014; Schwartz 1992). Schwartz “values theory” derives a limited number of values claimed to be present in all human cultures because they are grounded in the needs of individuals as biological organisms, the requirements imposed by the need for coordinated social interaction, and the needs of the survival and welfare of groups (Schwartz 1992, 2012; Schwartz et al. 2001). The SVS provides a means of identifying these values, and determining their relative importance in different groups. Comparability with our earlier studies was maintained using identical equipment and testing procedures.

## Materials and methods

### Participants

With local ethical approval, new experiments were conducted in the Department of Psychology, University of Menoufia, Shibin El Kom, Egypt. Seventy participants (median age 19 years; range 18–23 years; 40 males), all with normal or corrected-to-normal visual acuity, naïve to oculomotor testing, were recruited from the student body of the University of Menoufia. All provided written, informed consent and procedures were performed in accordance with the ethical standards laid down in the Declaration of Helsinki (as modified 2004). Comparison groups were two of those reported previously (Knox and Wolohan 2014). Briefly, these consisted of 70 participants born and educated in China, studying in the University of Liverpool as overseas students (median age 22 years; 15 males) and 70 Caucasian (i.e., white British) participants (median age 23 years; 27 males); these will be referred to throughout as “white British”, WB, participants.

Egyptian participants completed the Schwartz Value Survey (SVS) in Arabic. The SVS is a 57-item questionnaire from which ten value scores are generated by appropriate grouping of items. Individuals rate each item for its importance as a guiding principle in their life on a Likert scale of −1 (“against my values”) to 7 (“of supreme importance”; see Knox and Wolohan 2014, Supplementary information, for examples). The data were cleaned and processed in accordance with the instructions in the SVS User’s Manual (Schwartz 2009). The mean of each participant’s ratings across all items was calculated to provide the mean rating score; this was then subtracted from each individual item rating for that participant, correcting for individual response bias. The ten value scores were then computed and group mean scores for each value were generated for each of the participant groups. The two comparison groups (Chinese and WB, Knox and Wolohan 2014) had completed the Mandarin and English versions of the SVS (respectively).

### Apparatus and stimuli

Horizontal eye movements were recorded binocularly with the same miniaturised head-mounted infrared saccadometer (Ober Consulting, Poland) used in the previous experiments. This samples infrared reflectance signals at 1 kHz, and low-pass filters them at 250 Hz with 12-bit resolution. The device incorporates three low-power red lasers projecting red 13-cd/m^2^ target spots subtending approximately 0.1°, in a horizontal line, centrally and at 10° to left and right of centre. As the stimuli move with the head, participants were not head-fixed; as in the previous experiments, they sat in a comfortable position approximately 1.5 m in front of a near-white surface. Eye movement parameters and waveforms were stored for offline download and analysis.

### Procedures

After completing the Arabic version of the 57-item Schwartz Value Survey, participants were exposed to 4 runs of 200 trials, two of which were composed of gap trials (gap duration 200 ms), and two of which were overlap runs. The order of runs was counterbalanced across participants. Prior to each run, each component of the task was demonstrated to the participants. In gap trials, after a randomised fixation period of 1–2 s, the central fixation target was extinguished 200 ms prior to the appearance of the saccade target, presented randomly 10° either to the right or left. In overlap trials, the central fixation target remained illuminated throughout the trial. Again, after the randomised fixation period, the saccade target appeared. Regardless of trial type, participants were instructed to saccade to the eccentric target as soon as it appeared, pause, blink, and then return their gaze position to the centre in preparation for the next trial.

### Analysis

Saccade data were collated in MS Excel. For each participant, median saccade latency was calculated for gap and overlap tasks, the distribution of latency plotted, and the percentage of ES (saccades with a latency in the range 80–130 ms; %ES) out of all saccades in the latency range 50–500 ms calculated. This latency range and the express latency range were used for consistency with our earlier experiments. Participants in whom, in overlap conditions, %ES was 30% or greater, were classified as ESMs. For convenience, the other participants will be referred to as “normal”. Saccade latency was summarised using the intersubject mean of individual median latencies. To adequately summarise the latency distribution, average percentage distributions of latency were calculated. Each participant’s individual raw distribution was first converted to a percentage distribution. For each 10 ms bin, the mean (± 95% CI) was calculated across all participants. When plotted, this provided the average distribution. A line was plotted through each bin value to show the shape of the distribution. While no mathematical smoothing procedure was used, drawing a line does have the effect of visually smoothing the shape of the plot compared to plotting bars. Supplementary Fig. 1 illustrates the difference between plotting histogram bins and using a line plot. The resulting plot has the same shape as a pooled histogram, but the accompanying information about underlying variability (in the form of the 95% CI’s) demonstrates the degree to which this shape reflects the underlying data. Statistical analysis was conducted using SPSS. Analysis of variance (ANOVA) was used for multiple comparisons (with detailed designs and appropriate post hoc tests as noted in the results), and the Pearson chi-square test used for testing proportions.

## Results

**Fig. 1:**
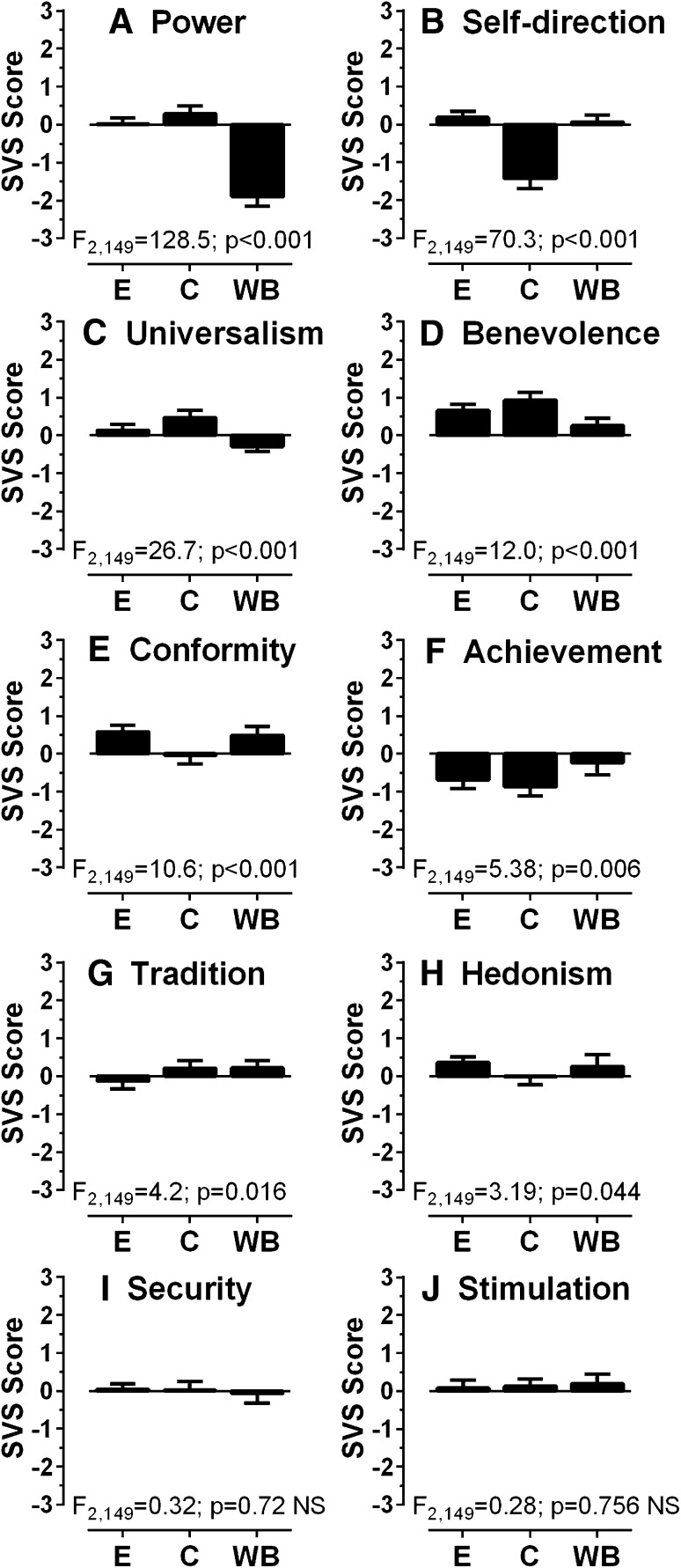
Plots of Schwartz Value Survey results. Mean ± 95% CI value scores for each of the ten “values” for each participant group are plotted (**e** Egyptian participants, *N* = 70; **c** Chinese participants, *N* = 38; **b** Caucasian (white British) participants, *N* = 42). Group scores for each of the ten Schwartz values were compared with a one-way ANOVA, and organised in order from highest *F* ratio (**a**) to lowest (**j**). Statistically significant results were observed for all values except “Security” and “Stimulation”

The pattern of the ten Schwartz values for the Egyptian participants is shown in Fig. 1 and compared to the values for Chinese and WB groups which we have published previously (Knox and Wolohan 2014). Using a 3 (group) × 10 (value) repeated-measures ANOVA, we found that both value (*F*_9,1341_ = 40.54, *p* < 0.001, *ηp* ^*2*^ = 0.21) and group (*F*_2,149_ = 19.40, *p* < 0.001, *ηp* ^*2*^ = 0.21) had a statistically significant effect on value scores and there was a significant interaction between them (*F*_18,1341_ = 24.32, *p* < 0.001, *ηp* ^*2*^ = 0.25). Given this, we compared group means for each of the ten values using simple main effects analysis. All values except “Stimulation” (Fig. 1j) and “Security” (Fig. 1i) yielded significant results, and for the eight significant values, a post hoc test (Tukey HSD) was used to examine the differences between the groups. What emerged was a heterogeneous pattern. For “Conformity” (Fig. 1e) and “Self-direction” (Fig. 1b), the Chinese group differed significantly from both the WB and Egyptian groups (*p* = 0.001 or less), who did not differ from each other for these values. For “Achievement” (Fig. 1f) and “Power” (Fig. 1a), it was the WB group that differed significantly from the Chinese and Egyptian groups (*p* = 0.01 or less), who were similar to each other. For “Universalism” (Fig. 1c) and “Benevolence” (Fig. 1d), the three groups differed from each other (*p* = 0.03 or less). For “Tradition” (Fig. 1g), the Egyptian group differed from both the Chinese and WB groups (*p* = 0.02 or less), with no difference between Chinese and WB groups. Finally, for Hedonism (Fig. 1h), the Egyptian group differed significantly from the Chinese group (*p* = 0.01) only, and the Chinese and WB groups did not differ.

**Fig. 2:**
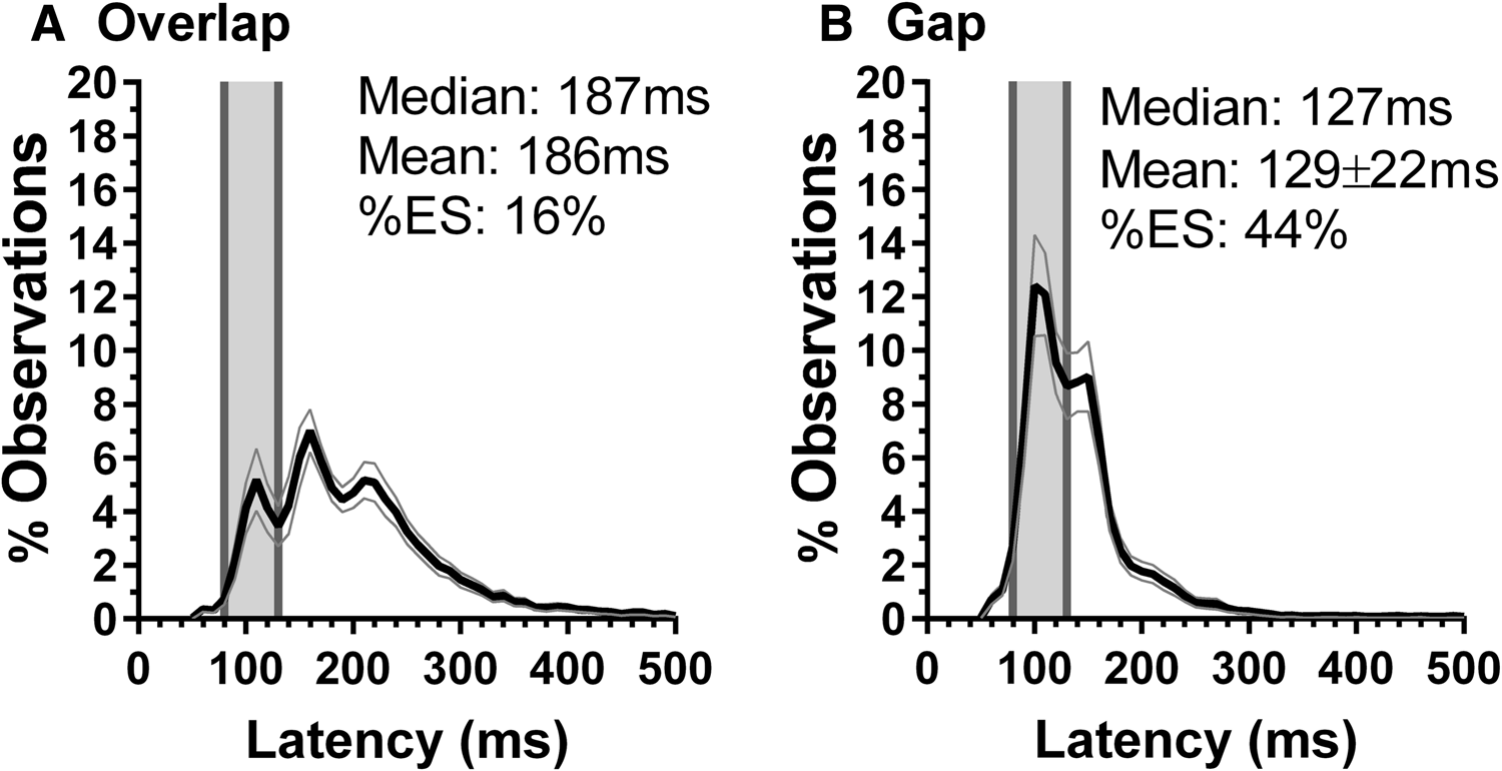
Average distribution histograms (bin means: black curve; ± 95% CI: grey curves) for saccade latency for all 70 Egyptian participants. Bin width = 10 ms. Vertical grey region shows the express saccade (ES) range (80–130 ms). Mean and median saccade latency, and the overall percentage of ES are shown. **a** Data from overlap tasks (fixation target visible when saccade target appears); **b** Gap tasks; gap duration = 200 ms

The intersubject mean (± SD) of Egyptian participant median saccade latency was 186 ± 36 ms for overlap tasks and 129 ± 22 ms for gap tasks. The mean gap effect (the difference between overlap and gap latency) was 58 ± 27 ms. Gender had a little influence on latency, although it tended to be lower in males. However, this only reached statistical significance in gap (males 123 ± 18 ms; females 133 ± 24 ms; *t* = 2.1, *p* = 0.04) and not in overlap conditions (males 178 ± 34 ms; females 193 ± 37 ms; *t* = 1.7, *p* = 0.09, Bonferroni corrected). The overall average distributions for overlap and gap tasks for the group of 70 Egyptian participants are shown in Fig. 2. The overlap distribution consists of three clear modes: an express mode (peak at 110 ms), a fast regular mode (peak 160 ms), and a regular mode (peak 210 ms). The lower overall mean latency in gap tasks was reflected in a much narrower distribution; while in the overlap distribution, the 90–270 ms bins all reached > 2% observations, this range only extended from 80 to 180 ms in the gap distribution. In the gap distribution (Fig. 2b), the express mode was the clearest (peak 100 ms) with a second peak at 150 ms. The proportion of anticipatory responses (those occurring in the 50–80 ms range, below the minimum limit used for identifying ES) was uniformly low. In gap tasks, the mean proportion of saccades with latency in this range was 1.85 ± 1.54%; in overlap tasks, it was 0.73 ± 0.75%.

**Fig. 3:**
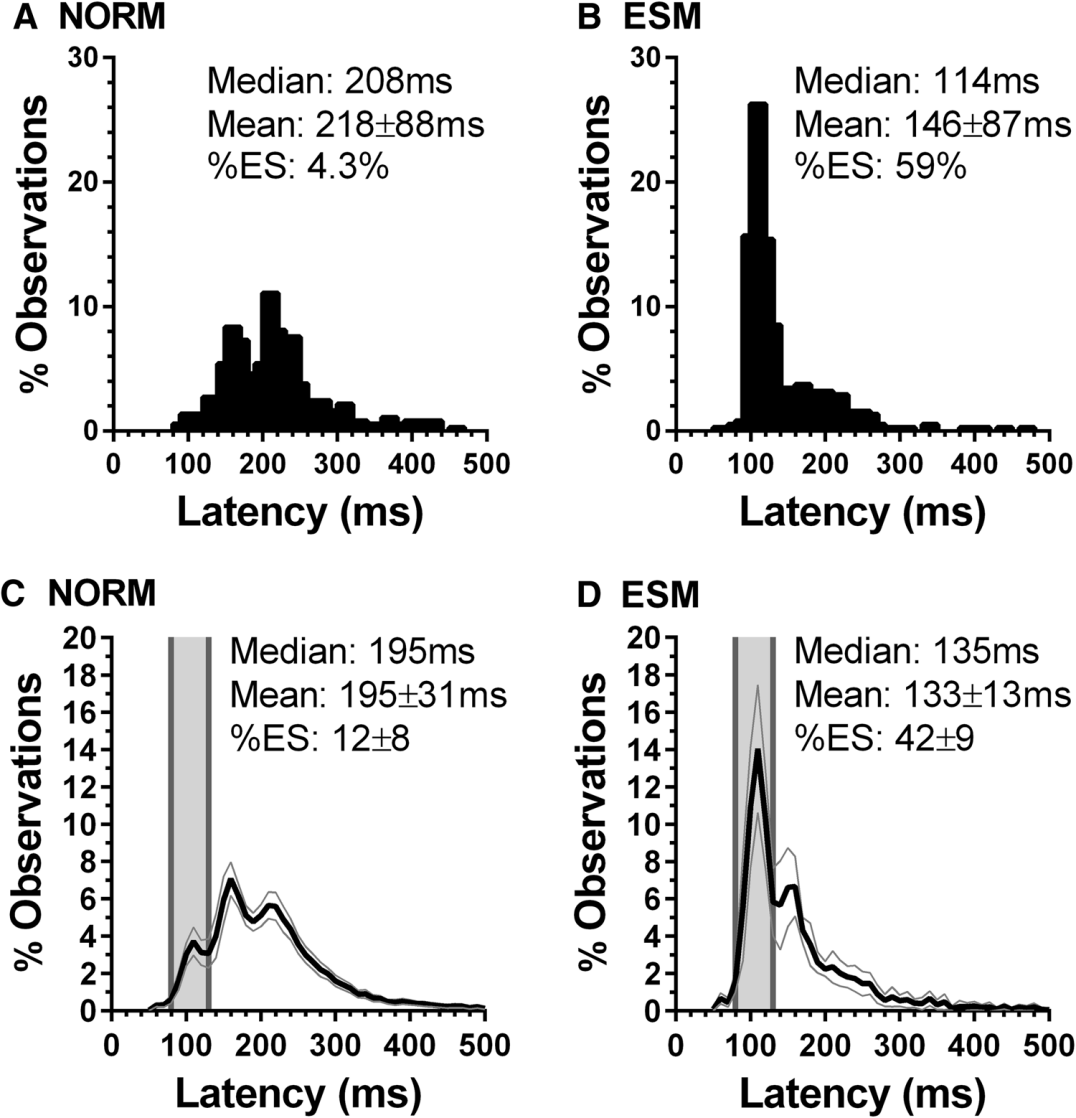
Individual example distributions of saccade latency in overlap tasks for a non-ESM (“normal”) participant (**a**) and an ESM (**b**). Mean/median latency and the percentage of ES (%ES) are also shown. **c** and **d** Average overlap distributions for 60 normal and 10 ESM participants

Gender had no statistically significant effect on the %ES in either gap (males 48 ± 21%; females 41 ± 18%; *t* = 1.5, *p* = 0.14) or more critically in overlap conditions (males 16 ± 15%; females 16 ± 13%; *t* = 0.05, *p* = 0.96). Of a total of 70 participants, 10 (14%) met the criterion of a minimum of 30% ES in overlap conditions. Typical individual distributions from overlap tasks for a normal and ESM participant are shown in Fig. 3 (a and b respectively). Note the prominent express peak in the ESM distribution. The mean proportion of express saccades for the 10 ESMs was 42 ± 9% compared to only 12 ± 8% for the normal participants (*t* = 10.8; *df* = 68; *p* < 0.001).

**Fig. 4:**
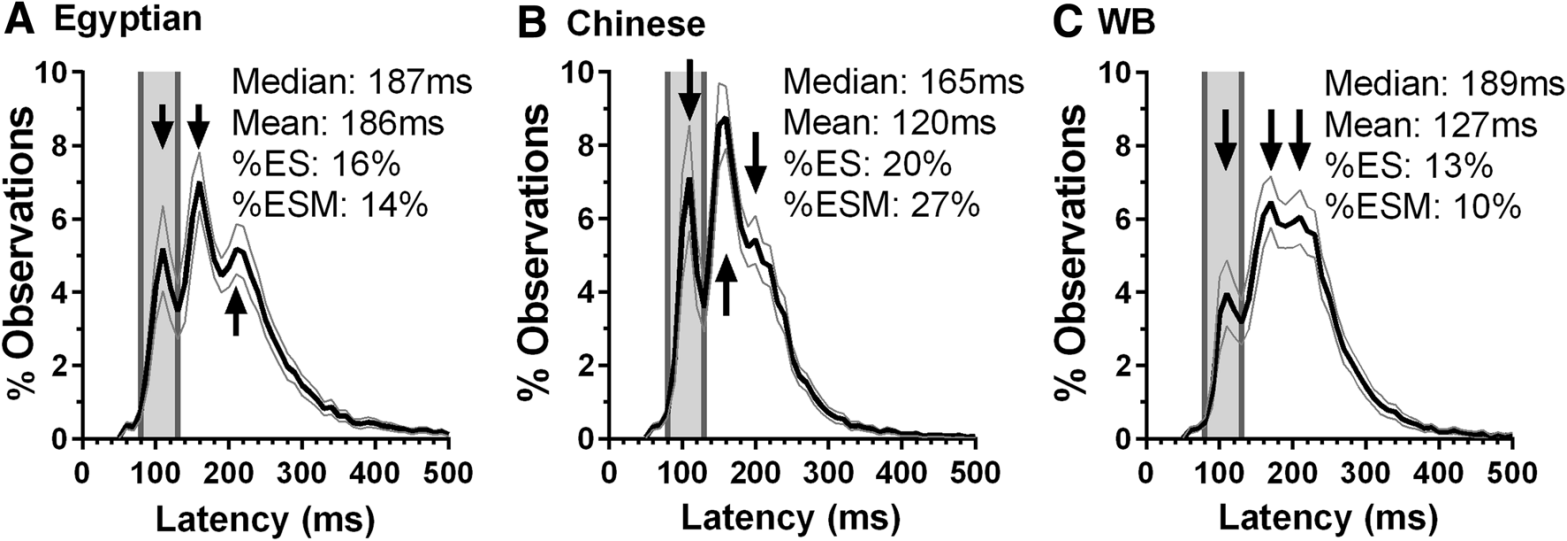
Comparison of average overlap distributions for all participants in each of the three groups. **a** Egyptian participants from the current study (replotted from Fig. 1). **b**, **c** Data from Knox and Wolohan (2014) for Chinese and WB participants respectively. The vertical black arrows indicate the three modes observed in the distribution from each group

We compared the new Egyptian data set with the saccade data from groups of 70 Chinese and 70 WB participants (Knox and Wolohan, 2014). Figure 4 illustrates average distributions for overlap tasks (the task which differentiated between ESM and normal participants) from the three groups. As noted above for the new data set, three modes could be distinguished in these distributions also. The earliest was the express mode (comprising express saccades). In all three distributions, this was centred on 110 ms (Fig. 4, most leftward arrow). The second mode, which was most obvious in the Egyptian and Chinese participant distributions (Fig. 4a, b, respectively), was at 160 ms. In the WB sample (Fig. 3c), there was a less obvious peak at 170 ms. There was a third mode which was most obvious in the Egyptian distribution and centred at 210 ms (Fig. 4a). This corresponded to a third mode in the other two distributions at 200 ms (Fig. 4b) and 210 ms (Fig. 4c).

A repeated-measures ANOVA using the participants’ individual median saccade latencies was run, treating group as a between-subjects factor, and task type (gap, overlap) and gender as within-subjects factors. While task type had a highly statistically significant effect (*F*_1,70_ = 687; *p* < 0.0001), the influence of gender, while still statistically significant in this analysis, was less marked (*F*_1,70_ = 10; *p* = 0.002). Neither the task type × gender interaction nor the group × gender interaction was statistically significant (*F*_1,70_ = 1.0, *p* = 0.29 and *F*_2,70_ = 1.5, *p* = 0.24, respectively; see Supplementary Table 1). The result for group was statistically significant (*F*_2,70_ = 10; *p* < 0.001); post hoc testing (Tukey HSD) suggested that while there was no significant difference between the WB and Egyptian groups, both differed from the Chinese group.

The mean %ES in each group varied, with the mean %ES for the Egyptian group (16 ± 3%) being intermediate between that observed for the Chinese and WB groups (20 ± 16% and 13 ± 12%, respectively). When tested with a one-way ANOVA, the result was statistically significant (*F*_2,207_ = 5.45; *p* = 0.005); post hoc testing revealed a significant difference between %ES in the Chinese and WB groups (Tukey HSD, *p* = 0.003), but not between Chinese and Egyptian (*p* = 0.22) and Egyptian and WB (*p* = 0.23).

An ANOVA similar to that run for latency, but now using the %ES, with group as a between-subjects factor, and task type (gap, overlap) and gender as within-subjects factors, demonstrated that again task type and gender had a statistically significant effect (*F*_1,70_ = 510, *p* < 0.001 and *F*_1,70_ = 11, *p* = 0.001, respectively) with no interaction between them (see also Supplementary Table 1). Group was also statistically significant (*F*_2,70_ = 4.2; *p* = 0.018); there was no interaction between group and gender (*F*_2,70_ = 0.3; *p* = 0.76). Post hoc testing again demonstrated a significant difference in %ES between Chinese and WB groups (*p* = 0.014), but not Chinese and Egyptian (*p* = 0.083) and Egyptian and WB groups (*p* = 0.641).

**Fig. 5:**
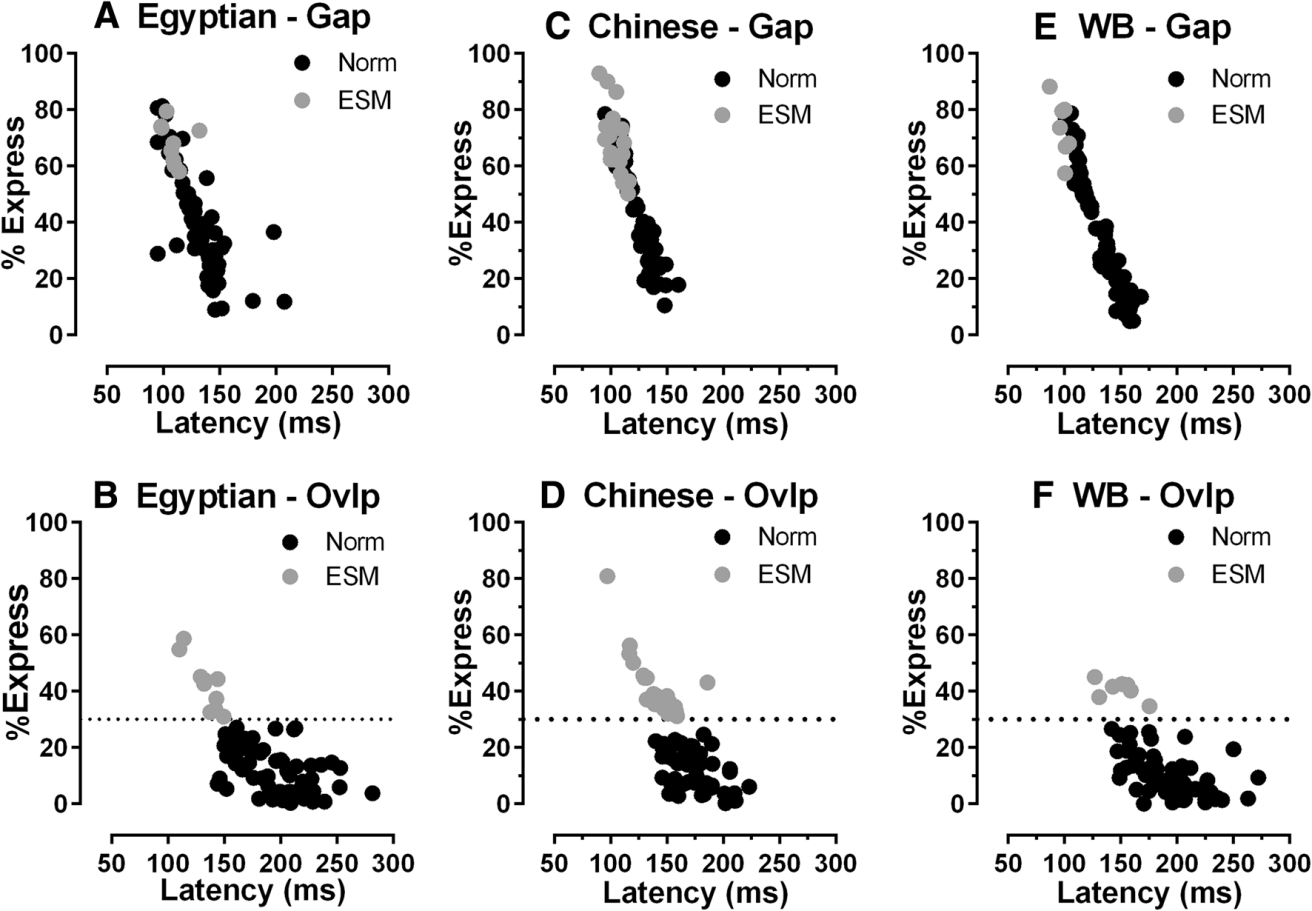
Express saccades and median saccade latency. The percentage of express saccades (%Express) is plotted against median saccade latency in gap (**a**, **c**, **e**) and overlap (**b**, **d**, **f**) conditions for each individual in the three participant groups (Egpytian **a**, **b**; Chinese **c**, **d**; WB **e**, **f**). ESMs filled grey circle denotes (participants with > 30% express saccades in overlap conditions); filled black circle denotes norm (non-ESM participants). The horizontal line in overlap plots (**b**, **d**, **f**) illustrates the 30% criterion above which participants were defined as ESMs

Plotting the %ES against the median saccade latency for both gap and overlap conditions, for these three groups of participants, revealed both similarities and differences (Fig. 5). In gap conditions, latency was more variable in the Egyptian group (minimum 95 ms, maximum 208 ms; coefficient of variation, CoV, 17%) than in the other two (Chinese: minimum 90 ms, maximum 160 ms, CoV 14%; WB 87 ms, maximum 168 ms, CoV 15%); some of the participants with the highest %ES were actually normal (non-ESM) participants. In overlap conditions, the mean intersubject latency for Egyptian participants (187 ± 36 ms) was similar to that observed in the WB group (189 ± 31 ms), but longer than that in the Chinese group (165 ± 25 ms). Figure 5 revealed that, in spite of a slightly higher proportion of ESMs, in the Egyptian participants, in overlap conditions, the range of individual latencies was wider with a tail that extended to longer latencies (maximum 282 ms), similar to the WB group (maximum 272 ms) and different to the Chinese group (maximum 190 ms). This difference was also reflected in the magnitude of the gap effect. This was smallest in the Chinese group (45 ± 17 ms) compared to the other two groups (Egyptian 58 ± 27 ms; WB 61 ± 27 ms).

Across the three groups of 70 participants, the proportion of ESMs was different. Compared to the Egyptian participants (10/70, 14%, were ESMs), 7/70 (10%) WB participants and 19/70 (27%) Chinese participants were ESMs. When tested with a Pearson chi-square test, the difference in proportions of ESMs between the three groups was statistically significant (*p* = 0.02). Post hoc comparisons (Bonferroni corrected) indicated that the Chinese group significantly differed from the WB group (*p* = 0.009), but not from the Egyptian group (*p* = 0.061). The WB and Egyptian groups did not differ significantly (*p* = 0.43).

## Discussion

Our aim in the present experiment was to collect saccade data on a large group of participants who were independent of, and distinct from, the groups we had examined previously. Across a number of studies, we have used data from large groups to examine patterns of saccade latency, investigating and comparing populations beyond those routinely sampled in saccade studies (and many other types of experiment; Henrich et al. 2010). In particular, we wished to establish the proportion of ESMs in this third group of participants.

Using the Schwartz Value Survey (SVS), we demonstrated that the new group of participants, who were geographically and ethnically distinct to the groups tested previously, were also culturally independent. By this, we simply mean that, at least based on the SVS scores, there was no evidence that our Egyptian participants were similar to the groups which we had tested previously. The SVS is designed to capture certain aspects of a culture, i.e., the relative importance of different values. Previously, we used it to demonstrate cultural differences between Chinese participants whose primary cultural exposure was within the UK (they had been born and educated in the UK) and Chinese participants from mainland China (Knox and Wolohan 2014). While the UK Chinese sample was culturally similar to a group of Caucasian (white British; WB) participants, as a group, they had the same high proportion of ESMs and identical saccade performance to the mainland Chinese group. Here, the SVS data showed marked differences between the three groups which we compared (Fig. 1).

We found that of the new Egyptian group 14% (10/70) were ESMs. This contrasts with what we found in three separate experiments with Chinese participants in which there were consistently high proportions of ESMs (29% Amatya et al. 2011; 22% Knox et al. 2012; 27% Knox and Wolohan 2014). In a group of 70 WB participants, 10% were ESMs (Knox and Wolohan, 2014). We have discussed the definition of ESMs and the main criterion used (> 30% of saccades in overlap conditions with latency in the ES range) extensively previously (Amatya et al. 2011; Knox and Wolohan 2014). Beyond the simple numerical criterion, what is striking is the clear express peak in the overlap latency distributions which is consistently observed in both individual ESMs (e.g., Fig. 3b) and the average distributions (Fig. 3d).

Simply, in terms of the proportion of ESMs, the post hoc analysis of the Chi-square test demonstrated that while that this was similar in the WB and Egyptian groups, it fell just short of demonstrating a statistically significant difference between the Egyptian and Chinese groups. It remains a possibility that the proportion of ESMs in the Egyptian group was in reality intermediate between the WB (low proportion of ESMs) and Chinese groups (consistently higher proportion of ESMs). As shown in Fig. 5, in terms of overall saccade performance, the Egyptian group exhibited both similarities and differences compared to the other groups. In addition to the slightly higher proportion of ESMs, in overlap conditions, there were Egyptian ESMs producing proportions of ES > 50%, similar to Chinese participants, but different to the WB group. On the other hand, examining the normal (non-ESM) participants in each group suggested that there were more participants in the Egyptian group who had relatively long median saccade latencies (> 230 ms) in common with the WB group. None of the Chinese participants, in overlap conditions, exhibited median latencies this long.

Drawing firm conclusions about these detailed difference in performance, ultimately involving a relatively small sample of individuals, is made difficult by differences in the composition of the three groups of participants. The Egyptian group was slightly younger than the other two, and this could have a bearing on the proportion of ESMs. In our original study on Chinese participants, a wider range of ages were tested than was the case in a number of our subsequent studies (including the current study). Most ESMs were aged less than 30 years (Amatya et al. 2011). Gender had little influence on either the latencies that we observed in the current study, nor did it affect the %ES in overlap conditions, the critical condition used to identify ESMs. Across the groups, latency tended to be lower and %ES higher in male participants, although the differences were small in absolute terms (see Supplementary Table 1). It was the Chinese group which demonstrated the most marked gender differences, and this was the most unbalanced of the groups in terms of gender (79% of participants were female). However, given the direction of the effect of gender on latency and %ES, this oversampling of females does not explain the higher overall proportion of ESMs in this group.

With regard to saccade performance, more generally, the new group of participants exhibited the familiar gap effect (the difference in median latency between gap and overlap conditions) on saccade latency of a similar magnitude to that which has been reported previously (Kingstone and Klein 1993; Saslow 1967b). Average latency distributions also exhibited a marked multimodality. The detection of several modes in saccade latency distributions has been reported for both gap and overlap saccade tasks (Fischer et al. 1993; Gezeck et al. 1997). What is of interest is that using different approaches, the timing of the modes appears to be highly stereotyped. Gezeck et al. (1997), using a statistical method, reported that the most consistently observed modes had latency ranges of 90–120 ms (the express mode), 135–170 ms (the fast regular mode), and 200–220 ms (the regular mode). These timings are very similar to what we observed in our average distributions across all three participant groups. It may be of significance that to identify these distribution patterns, Gezeck et al. (1997) accumulated a large data set drawn from 170 experimental subjects. Whether an individual’s distribution exhibits more than one mode in a given set of experimental conditions appears to be highly variable across participants. Thus, Gezeck et al. (1997) observed more than one mode in 45% of individual distributions. However, identifying modes in distributions, even if present, is difficult if only small numbers of trials per participant are available; sparse distributions will lack sufficient detail. Details are also lost by inappropriate clumping of data (Noorani and Carpenter 2011). This relates to the issue of how to adequately summarise distributions across participants.

In experiments with small participant numbers (e.g., < 5), individual histograms have been often been presented (Findlay 1981; Jüttner and Wolf 1992; Kingstone and Klein 1993). However, this makes detecting all but the most obvious patterns difficult, as well as raising issues of how representative these are. For some experiments, this may not matter. In many early experiments on the gap effect, the aim was simply to see if there was an early mode representing express saccades in gap conditions as had been reported for non-human primates. With larger datasets, presenting many individual distributions makes distinguishing general patterns difficult because of the intersubject variability that is one of the intriguing features of patterns of saccade latency (Amatya et al. 2011; Gezeck et al. 1997). While some features may be relatively easily identified (e.g., the express peak in the distributions of ESMs; Amatya et al. 2011; Knox et al. 2012—see supplementary information), simply presenting many individual histograms risks obscuring as much as it reveals. Pooled histograms, in which data from a number of participants (and occasionally conditions) are combined and then calculating a single distribution, are relatively common (Delinte et al. 2002; Dickov and Morrison 2006; Heeman et al. 2017). This results in the identical shape of distribution to the average distributions which we have used, but provides no information as to how adequately the shape of the distribution represents the underlying data. Average distributions provide a useful alternative in both allowing the identification of modes and their timing, and providing information on how well the shape of the distribution represents the underlying data (by also plotting 95% confidence intervals for each bin). Using this approach, with the stimulus conditions we have used, with large numbers of trials per participant per condition and large groups of participants, three modes are identifiable in overlap conditions. The express and fast regular modes are the clearest (Fig. 4), particularly for Egyptian and Chinese groups, driven by the presence of higher proportions (at 14 and 27%, respectively) of ESMs compared to the WB group. However, a third mode (the slow regular mode; Gezeck et al. 1997) also seems to be present at the same latency in all three distributions.

We conducted additional analysis to investigate whether multimodal distributions could be an artefact of our procedures, particularly those involved in constructing average histograms (Supplementary Fig. 1). Taking a real unimodal distribution from a single participant (Supplementary Fig. 1A), we found that when multiple distributions were averaged with the peaks of the individual distributions separated into two groups positioned around 160 and 210 ms, the resulting average distribution was unimodal (Supplementary Fig. 1c). If the groups were much further apart (160 and 240 ms), we could produce a bimodal average distribution (Supplementary Fig. 1D), but the second peak was the larger of the two, something we observed in none of our participant groups.

Across a number of experiments, we have observed multimodal saccade latency distributions, and been able to identify ESMs without difficulty. We have used both randomised fixation times and randomised target direction (although for only two target positions). High levels of predictability are known to encourage express saccade production; with only two potential target positions, this might encourage at least bimodal distributions. Recently, Marino et al. (2015) demonstrated that target luminance had a clear effect on the shape of saccade latency distributions in non-human primates (as well as generally increasing the proportion of ES; Marino and Munoz 2009). At low target luminance, just above detection threshold, saccade latency was distributed unimodally. As target luminance increased, the shape noticeably changed with the appearance of an early mode. Even several log units are above detection threshold, there appeared to be changes in the shape of the distribution, although the timing of the two main modes appeared to be relatively fixed. Our fixation and saccade targets were bright laser spots, several orders of magnitude above detection threshold (although we did not measure this formally). Notwithstanding the randomisation of fixation time and direction, the combination of stimulus conditions which we have used may have facilitated both the identification of ESMs and the multimodality observed across groups.

With consistent equipment and procedures, the patterns of saccade latency which we have observed and the characteristics of ESMs have been consistent across several large participant groups. It is unclear whether the three latency modes that we have confirmed (express, fast regular and regular; Fischer et al. 1993; Gezeck et al. 1997) reflect the neurophysiological structure (i.e., distinct anatomical pathways) or function (distinct processes such as fixation disengagement or attentional deployment) of the saccade system, although these are not mutually exclusive. For visually guided saccades, most attention has historically focussed on the importance of the distinction between the express and other modes in gap conditions, with explanations primarily provided at the level of the superior colliculus (Dorris et al. 1997; Edelman and Keller 1996). There has been much less discussion of what might produce distinct populations of fast regular and regular saccades. It may be that this aspect of the saccade latency distributions is primarily dependent on mechanisms beyond the colliculus (e.g., the balance between frontal and parietal networks).

Further investigation of patterns of saccade latency in large participant groups drawn from different populations would be of value. Given the relative ease with which saccade parameters can be extracted and analysed, this would allow a comprehensive analysis of those functional aspects of behaviour that are relatively fixed and the factors (biological, psychological, social, and environmental) to which they relate.

## Electronic supplementary material

Below is the link to the electronic supplementary material.

Supplementary material 1 (DOCX 16 kb)

Supplementary material 2 (DOCX 80 kb)
