# Supplementary material for: Express saccades in distinct populations: east, west, and in-between
Source: Exp Brain Res. 2017 Sep 27;235(12):3733–42. doi: 10.1007/s00221-017-5094-1 (PMC5671528; doi:10.1007/s00221-017-5094-1)
Supplement: Supplementary file 1 — Supplementary material 1 (DOCX 16 kb) [file 221_2017_5094_MOESM1_ESM.docx]

|  | **Gap** | | | |  | **Overlap** | | |
| --- | --- | --- | --- | --- | --- | --- | --- | --- |
|  | **WB** | **Chinese** | **Egyptian** | |  | **WB** | **Chinese** | **Egyptian** |
| **%ES** | | | |  | |  | | |
| **Male** | 45.67(23.88) | 55.53 (22.75) | 48.16 (20.78) | |  | 12.74 (12.67) | 27.85 (17.15) | 16.47 (14.82) |
| **Female** | 39.11(21.74) | 48.01 (20.24) | 41.21 (17.56) | |  | 12.39 (11.35) | 18.21 (15.24) | 16.32 (12.51) |
| **Latency (ms)** | | | |  | |  | | |
| **Male** | 123 (21) | 116 (16) | 123 (18) | |  | 193 (33) | 149 (25) | 178 (34) |
| **Female** | 130 (18) | 121 (16) | 133 (24) | |  | 187 (30) | 169 (24) | 193 (37) |

Supplementary Table 1. Group intersubject mean (SD) %ES (upper) and latency (lower) for the three comparison groups split by gender.
