# Supplementary material for: Express saccades in distinct populations: east, west, and in-between
Source: Exp Brain Res. 2017 Sep 27;235(12):3733–42. doi: 10.1007/s00221-017-5094-1 (PMC5671528; doi:10.1007/s00221-017-5094-1)
Supplement: Supplementary file 2 — Supplementary material 2 (DOCX 80 kb) [file 221_2017_5094_MOESM2_ESM.docx]

**Supplementary Figure 1.**

A. Individual histogram for a single nonESM participant in overlap conditions; data were distributed unimodally, with a peak at 160ms. Number of trials: 400. Mean and median latency are shown. B. The same histogram, but drawing a line through each bin value rather than plotting histogram bins. No other smoothing procedure was used. C. The effect of averaging as employed in this study. Base distributions shown in A were jittered over two time windows centred on 160ms (five peak positions: 140ms, 150ms, 160ms, 170ms,180ms) and 210ms (190ms -230ms). This was done for seven sets of distributions (ie 35 around 160ms, 35 around 210ms; total 70). These were then averaged: the mean (±95% CI) was calculated for each bin. The black line plots the mean bin values, with the grey lines illustrating the 95% CI. The resulting average distribution is unimodal. D. The same procedure, with the same base distribution (A), but centred on 160ms and 240ms (220ms-260ms). The resulting distribution is bimodal, with the second peak the larger of the two.
